# Prevalence of Health App Use Among Older Adults in Germany: National Survey

**DOI:** 10.2196/mhealth.8619

**Published:** 2018-01-23

**Authors:** Peter Rasche, Matthias Wille, Christina Bröhl, Sabine Theis, Katharina Schäfer, Matthias Knobe, Alexander Mertens

**Affiliations:** ^1^ Department of Mechanical Engineering Institute of Industrial Engineering and Ergonomics RWTH Aachen University Aachen Germany; ^2^ Department of Orthopaedic Trauma University of Aachen Medical Center RWTH Aachen University Aachen Germany

**Keywords:** telemedicine, Germany, mobile applications, smartphone, aged

## Abstract

**Background:**

Health apps are increasingly becoming an integral part of health care. Especially in older adults, the self-management of chronic diseases by health apps might become an integral part of health care services.

**Objective:**

The aim of this explorative study was to investigate the prevalence of health app use and related demographic factors, as well as health status among older adults in Germany.

**Methods:**

A nationwide postal survey was conducted. Of the 5000 individuals contacted, a total of 576 participants completed this survey. On the basis of their self-indicated assignment to one of the three predefined user groups (health app users, general app users, and nonusers of apps), participants answered various questions regarding app and health app use, including frequency of use and number of installed apps, demographic factors, and health status.

**Results:**

In total, 16.5% (95/576) used health apps, whereas 37.5% (216/576) indicated only using general apps, and 46.0% (265/576) reported using no apps at all. The number of installed health apps was most frequently reported as between 1 and 5 apps per participant, which were usually used on a weekly basis. The most frequently cited type of health apps were exercise-related ones. Individuals using health apps were found to be younger (MeanmHealth 66.6, SD 4.7) and to have a higher level of technical readiness compared with general app users and nonusers of apps (adjusted odds ratio, AOR=4.02 [95% CI 2.23-7.25] for technical readiness, and AOR=0.905 [95% CI 0.85-0.97] for age). The most frequently mentioned sources of information about apps within the group of health and general app users were family and friends. Identified barriers against the use of health apps were a lack of trust, data privacy concerns, and fear of misdiagnosis.

**Conclusions:**

Health apps are already used by older adults in Germany. The main type of apps used are exercise-related ones. Barriers to and incentives for the use of health apps and associations with health status and users’ demographics were revealed.

## Introduction

The number of households with individuals older than 75 years will double by 2050. Fifty percent of the population of Germany will then be older than 50 years and 12% older than 80 years [1]. In this context, aging is often associated with a decline in quality of life (QoL) because of factors such as limited mobility and autonomy and the appearance of various chronic diseases [2]. By providing older adults with suitable health apps, they might be enabled to monitor and manage health in an independent and self-determined manor [3-7]. Smartphones have a large number of sensors able to measure and track vital parameters as well as other health-related data [3]. Health apps analyze and process these data and could therefore provide integral support to health care services in the future [3,6,8]. There are numerous examples of such health apps that cover diseases such as diabetes or other chronic conditions as well as topics such as fall risk. To date, only a small number of health apps have been introduced into the health care market [9-12].

As health apps are a relatively new phenomenon, it is not yet known to what extent health apps are being accepted and used by older adults for personal health care. Initial studies into the use of health apps among the general population have been carried out in the United States and Hong Kong [13,14]. In the United States, 19% of smartphone owners had used at least one health app in 2012 already [14]. The study did not, however, measure the level of use among older adults within the sample. A more recent study revealed a use rate in 2015 of more than 50% within a sample of younger and older adults in the United States [15]. A different study in Hong Kong identified a use rate of about 20% among this population in 2016 [13]. The most frequently mentioned health apps in all these studies were exercise-related. Nevertheless, the question remains whether older adults are willing to integrate such communication-technology–based health care products into their daily lives. For them, this means that they would have to adapt to the new technology, especially as they are “digital immigrants” who might not be engaged with smartphones and related products [16].

To the best of our knowledge, we were the first to investigate this question by evaluating whether older adults in Germany have any health apps installed on their mobile devices, how many apps they have installed, and how often they use them.

### Aim of This Study

In this study, we investigated the prevalence of health apps among older adults in Germany, incentives for and barriers to their use, as well as which sources users rely on to gain information about health apps. We performed a postal survey questioning possession and use of health apps related to demographic factors and health status as measured by a health literacy scale and history of chronic diseases [17].

### Research Questions

In summary, our main research questions were as follows:

- Do older adults in Germany use health apps?
- What types of health apps are currently being used by older adults?
- What factors are associated to health app use?
- What sources do older adults rely on to retrieve information about apps?

## Methods

### Design

A postal survey was designed to investigate the aforementioned research questions. The survey was designed in German and provided for German-speaking older adults. A postal survey was used as it is a cost-effective way to reach older adults in a short period of time without any limitations on physical space [18,19].

On the basis of the research questions, the main purpose of the survey was to collect data about three different user groups that we would like to compare. The three predefined user groups we wanted to identify and compare were older adults who use health apps (“health app users”), those who use general apps but not health apps (“general app users”), and those who had never used an app (“nonusers of apps”). To identify these three groups, users were asked to state to which of them they belong.

### Characterizing Participants

To characterize users of health and general apps, more detailed questions about the number of installed apps and frequency of use, as well as health status, were asked.

#### Measuring Technology Readiness

Technology readiness was included as it might influence the use of modern information and communication technology, as well as the engagement with these products [20]. It is calculated based on 12 standardized items which are rated on a 5-point Likert scale (1=not correct, 5=fully correct). For positively formulated objects, the scale is converted so that a high point value corresponds with high technology readiness. Subsequently, the final score is calculated by mean value over all 12 items. The score therefore ranges between 1 point and 5 points [20].

#### Measuring Computer Literacy

Computer literacy was included as it identifies whether an individual has high or low experience in using computers [21]. Computers have been available for a much longer period of time and are more widely used than smartphones. We therefore wanted to determine whether high experience with computers might influence the use of health apps, as such individuals might have lower barriers to using this technology. The computer literacy scale questionnaire was used in a shorted version with 20 items. On the basis of a dichotomous coding of the responses (false=0, correct= 1), the score was calculated as over the sum of all items. The final score, therefore, ranges from 0 points up to 20 points, whereby a high score indicates high computer literacy [21].

#### Measuring Health Status

Health apps are a part of the field of consumer health, so it is interesting to investigate whether an association between health app use and health status exists [6]. Shen et al identified an association between history of chronic disease and health app use among the general population of Hong Kong [13]. We wanted to know whether this is the same for older adults in Germany. Participants were asked whether they suffer from chronic diseases and, if so, which type. Additionally, the health competency of participants was measured using an adapted version of the European Health Literacy Survey-47 (EU HLS-47), with 15 items [17]. Corresponding statements were evaluated on a 5-point Likert scale (1=not correct, 5=fully correct). Subsequently, the point values were converted into dichotomous values and summed [22]. Therefore, the final score ranges between 0 points and 15 points, whereby a high score indicates high health competency [17].

### Investigating Use of Health Apps

The main aim of this study was to determine whether older adults in Germany use health apps. Therefore, all participants were asked whether they use health apps or not. If participants indicated using health apps, they were sorted into the group of health app users. Besides the simple question of whether participants use health apps or not, the number of health apps installed on participants’ smartphones was also inquired. In addition, participants were asked how frequently they use their health apps and which type of health app they use in particular. As these figures alone are not meaningful, participants were also asked about the number of general apps installed and their usage frequency.

### Investigating Acceptance of Health Apps

A second goal of this study was to investigate the barriers to the acceptance of health apps. Both health app users and general app users were surveyed. The group of nonusers of apps was not considered in this question because the lack of experience with apps was assumed to be too influential. Both other groups (health app users and general app users) were asked what reasons they would personally argue against using health apps. In addition to a free text field, further answers were given. These reasons include privacy concerns, lack of self-confidence, fear of misdiagnosis, poor usability, and lack of confidence in the app. Multiple answers were possible for this question.

### Identifying Sources of Information About Apps

The third goal of this study was to identify different sources of information older adults use to find information about apps. Here too, only health app users and general app users were surveyed. The participants were able to select from different predefined answers the ones that fit them best. Available sources of information were family and friends, Internet, app store, magazines, television, and experts, with experts the least frequently cited source of information.

### Data Collection

Data were collected from July 2016 to September 2016. The questionnaire was printed and sent by postal mail. The survey was introduced as a study examining the effects of modern digital technology on the German health care system (see Multimedia Appendix 1).

All of the participants were informed about the duration of the survey, data storage, and the leading investigator. Each participant decided to take part in this survey voluntarily by sending back the completed questionnaire in a postage-paid envelope. No incentives were offered for participation.

The survey was tested properly by five independent examiners with regard to wording. The paper-based questionnaire included 109 items distributed over 15 different pages.

### Recruitment

The survey was addressed to 5000 older adults retrieved from the general German population by the external service provider Deutsche Post AG. Inclusion criteria for the selection by Deutsche Post AG were older than 60 years and residence in Germany. No exclusion criteria or screening questionnaires were applied.

All questionnaires were sent by post. The sampling procedure was nonprobabilistic, and respondents were self-selected based on their voluntary willingness to participate. This method of recruitment was chosen as the probability that participants with no or limited experience in the topic of apps would be reached by a Web-based survey is quite low [19]. Bech and Kristensen investigated differences in response rates regarding a potential future design of nursing homes. Their study revealed a higher response rate for a postal than a Web-based survey among older adults [23]. Hence, a postal survey was conducted in this study. Furthermore, this postal survey is an observational study targeting participants who use or do not use general and health apps. Recruitment via postal mailing, therefore, seems to be a suitable and cost-effective approach [18,19].

In total, N=5000 unique individuals were contacted by postal mail. However, only n=576 contacted older adults participated in the survey and completed the questionnaire. The participation rate is thus 11.52% (576/5000). Due to the method of data collection, the completion rate is unknown. On the basis of pretests, it can be stated that the average time required to complete the survey was around 45 min.

### Statistical Analysis

Data were analyzed using Statistical Package for the Social Sciences (SPSS) version 22 (IBM Corp). The associations of age, sex, education, technical readiness, computer literacy, health competence, and history of diagnosed chronic disease with health app possession were analyzed by logistic regression in a model with these variables mutually adjusted. The associations of age, sex, education, technical readiness, computer literacy, health competence, and multimorbidity (defined as the number of chronic diseases participants suffer from) with the different reasons for not accepting health apps and preferred sources of information were analyzed by logistic regression in separate models with these variables mutually adjusted. To compare general and health app users, we also calculated *t* tests for independent samples and chi-square statistics both at a significance level of .05.

### Ethics Statement

The ethics committee at RWTH Aachen Faculty of Medicine authorized this study and its ethical and legal implications in its statement EK236/16.

## Results

### Participants

In total, 576 individuals took part in this study. Mean age was 69.17 years (SD 5.76). Of the 576 participants, 280 (48.7%) were female. Some 10.1% (57/576) of the participants achieved primary education level, 57.3% (330/576) achieved secondary education level, and 32.5% (187/576) achieved tertiary education level. A total of 286 out of 576 participants (49.6%) use a smartphone and 132 out of these 286 smartphone users (46.2%) also use a tablet. Just 33 out of 576 participants (5.7%) use a tablet without an additional smartphone.

Depending on participants’ statement regarding health or general app use, they were divided into three user groups: users of health apps (n=95), users of general apps (n=216), and nonusers of apps (n=265). Table 1 shows detailed sociodemographic information for participants as sorted into the three different groups.

On average, users in all three groups reported at least one chronic disease per participant (Mean_mHealth_ 1.3 [SD 1.3], Mean_general_ 1.1 [SD 1.1], and Mean_nonusers_ 1.1 [SD 1.1]). The reported number of chronic diseases did not differ significantly between the three groups, *F*_1,309_=0.08, *P*=.78. Table 2 provides an overview of the different types of chronic diseases reported. All groups mentioned hypertension most frequently. Back pain, arthrosis, and diabetes were also frequently mentioned chronic diseases within all three groups (see Table 2). The only significant differences between the three user groups occurred for osteoporosis and back pain; health users did not mention osteoporosis at all, χ^2^_2_=7.1, *P*=.02, and back pain was reported most frequently by nonusers, followed by health app users and then general app users, χ^2^_2_=8.5, *P*=.01.

### Use of Health Apps

This section reports the findings relating to health app use. We focus especially on predictive factors to identify incentives for and barriers to getting older adults engaged with health apps.

Participants were asked how many general apps they have installed. The two groups (health app users and general app users) differ significantly, χ^2^_4_=24.7, *P*<.001. The most frequently cited number was “up to ten” for both health app users (37%, 35/95) and general app users (60%, 129/216). Additionally, participants were asked how often they use general apps. The most frequent answer within the group of health apps users was “daily” (73%, 69/95), the same as for the group of general app users (49%, 106/216). However, the two groups differ significantly for time of use, χ^2^_4_=17.6, *P*<.001. Table 3 reports the detailed frequencies.

**Table 1:** Participant demographics by user group.

Participant demographics		User group		
		Health app users	General app users	Nonusers of apps
**Sample**				
	Number of participants, n	95	216	265
**Age, years**				
	Minimum	61	60	61
	Maximum	82	80	90
	Mean (SD; years)	66.6 (4.7)	68.2 (5.1)	70.8 (6.1)
**Gender**				
	Male, n (%)	54 (56.8)	117 (54.2)	124 (46.8)
	Female, n (%)	41 (43.2)	98 (45.8)	141 (53.2)
**Education**				
	No education, n (%)	0 (0.0)	0 (0.0)	1 (0.3)
	Low level, n (%)	3 (3.1)	16 (7.4)	37 (14.0)
	Average level, n (%)	42 (44.2)	99 (45.8)	140 (52.8)
	High level, n (%)	49 (51.6)	95 (44.0)	78 (29.4)
	Other, n (%)	1 (1.1)	6 (2.8)	7 (3.5)
**Technical readiness**				
	Range (points)	2.17-5	1.67-4.83	2.17-5
	Mean (SD; points)	3.7 (0.6)	3.4 (0.6)	2.9 (0.6)
**Computer literacy**				
	Range (points)	2-18	3-18	1-18
	Mean (SD; points)	27.0 (3.9)	13.5 (3.7)	10.0 (5.1)
**Health competence**				
	Range (points)	3.5-15	5-15	3.5-15
	Mean (SD; points)	11.3 (2.8)	10.8 (2.6)	10.5 (2.9)

**Table 2:** Mean number and types of chronic diseases reported per group (multiple answers allowed).

Number and types of chronic diseases		User group			Significance
		Health app users	General app users	Nonusers of apps	
Number of chronic diseases, mean (SD)		1.1 (1.1)	1.1 (1.1)	1.3 (1.3)	*F*=0.08 (1,309), *P*=.78
**Types of chronic diseases, n (%)**					
	Hypertension	37 (38.9)	80 (37.0)	106 (40.0)	χ^2^_2_=0.4, *P*=.80
	Back pain	20 (21.1)	38 (17.6)	76 (28.7)	χ^2^_2_=8.5, *P*=.01
	Arthrosis	17 (17.9)	36 (16.7)	53 (20.0)	χ^2^_2_=0.9, *P*=.63
	Diabetes	15 (15.8)	22 (10.2)	41(15.5)	χ^2^_2_=3.3, *P*=.19
	Overweight	12 (12.6)	28 (13.0)	45 (17.0)	χ^2^_2_=1.9, *P*=.38
	Cardiovascular disease	8 (8.4)	24 (11.1)	42 (15.8)	χ^2^_2_=4.3, *P*=.11
	Respiratory disease	6 (6.3)	14 (6.5)	19 (7.2)	χ^2^_2_=0.1, *P*=.93
	Osteoporosis	0 (0)	7 (3.2)	16 (6.0)	χ^2^_2_=7.1, *P*=.02

**Table 3:** Number of installed general apps and time of use.

Number and frequency of use		User group		Significance
		Health app users	General app users	
**Number of installed general apps, n (%)**				χ^2^_4_=24.7, *P*<.001
	≤10	35 (36.8)	129 (59.7)	
	11-20	33 (34.7)	37 (17.1)	
	21-30	16 (16.8)	15 (6.9)	
	31-40	4 (4.2)	2 (0.9)	
	>40	3 (3.2)	4 (1.9)	
**Frequency of use, n (%)**				χ^2^_4_=17.6, *P*<.001
	Daily	69 (72.6)	106 (49.1)	
	Every 2-3 days	17 (17.9)	41 (19.0)	
	Weekly	4 (4.2)	15 (6.9)	
	Monthly	1 (1.0)	18 (8.3)	
	Never	0 (0)	31 (14.4)	

To examine the use of health apps in detail, we asked users how many health-related apps they have installed and how often they use them. The most frequently reported number of installed apps was “1-5 health apps” (85%, 81/95), followed by “6-10 health apps” (3%, 3/95), and just one participant reported having “11-15 health apps” (1%, 1/95). Health app users stated they have up to 20 general apps installed. Asked about the frequency of use, health app users most frequently mentioned weekly use (27%, 26/95), followed by monthly (22%, 21/95) and “every 2-3 days” (16%, 15/95), with just 10 participants claiming to use health apps on a daily basis (11%, 10/95). Comparing these frequencies with frequencies of general app use reveals a significant difference, *t*_88_=−13.54, *P*<.001. Health apps are less frequently used than general apps.

Users of health apps were also asked about the type of health apps they use (multiple answers allowed). The most frequently reported type were exercise apps (29%, 28/95), followed by apps for rating (26%, 25/95), first aid response (13%, 12/95), diabetes management (12%, 11/95), diagnosis (12%, 11/95), emergency passport (9%, 9/95), dairy (8%, 8/95), medication planner (7%, 7/95), and for contacting the physician (1%, 1/95).

Logistics regression revealed technical readiness and age to be associated with use of health apps (AOR=4.02 [95% CI 2.23-7.25] for technical readiness and AOR=0.905 [95% CI 0.85-0.97] for age). Sex, education, computer literacy, health competence, multimorbidity, and field of work were not associated with having health apps. Descriptive comparison showed that the mean age within the group of health application users is lower than within the group of general app users. The highest mean age was found for the group of nonusers of apps. The mean values of technical readiness show that health app users have the highest level of technical readiness followed by general app users and then nonusers (Mean_mHealth_ 3.7 [SD 0.6], Mean_general_ 3.4 [SD 0.6], and Mean_nonusers_ 2.9 [SD 0.6]).

### Acceptance of Health Apps

Participants were asked for reasons decreasing subjective acceptance of health apps. Hence, a more detailed picture is drawn of the barriers older adults might have toward using health apps. Table 4 shows that a lack of trust in health apps is the major barrier to using them.

Users of general apps mentioned significantly more reasons than health app users, *t*_310_=25.37, *P*<.001. The most frequently mentioned reason within both groups is a lack of trust, followed by data privacy concerns and fear of misdiagnosis. The two groups differ significantly in the case of poor usability, χ^2^_1_=4.8, *P*<.02 and a lack of self-confidence, χ^2^_1_=4.7, *P*=.03. Both reasons were more frequently mentioned by users of general apps than of health apps.

Analysis of association between the reasons against acceptance and demographic characteristics revealed different associations (see Table 5). A lack of trust is positively associated with technical readiness and computer literacy (AOR=1.74 [95% CI 1.18-2.58] for technical readiness and AOR=1.08 [95% CI 1.02-1.14] for computer literacy). Furthermore, data privacy concerns are positively associated with technical readiness and computer literacy (AOR=1.66 [95% CI 1.01-2.71] for technical readiness and AOR=1.11 [95% CI 1.03-1.21] for computer literacy). The fear of misdiagnosis is associated with decreasing age and increasing multimorbidity and technical readiness (AOR=0.92 [95% CI 0.85-0.99] for age, AOR=1.42 [95% CI 1.07-1.89] for multimorbidity, and AOR=1.94 [95% CI 1.01-3.73] for technical readiness). Poor usability was associated with increasing age and decreasing health competence and decreasing technical readiness (AOR=1.08 [95% CI 1.01-1.16] for age, AOR=0.8 [95% CI 0.69-0.92] for health competence, and AOR=0.41 [95% CI 0.21-0.78] for technical readiness). Finally, a lack of self-confidence was associated with decreasing health competence and technical readiness (AOR=0.66 [95% CI 0.53-.82] for health competence and AOR=0.32 [95% CI 0.13-0.77] for technical readiness).

### Sources of Information About Apps

Besides use and acceptance, we also investigated where participants acquire information about health apps. Table 6 shows which sources participants rely on.

Health app users use significantly more sources of information about apps than users of general apps (Mean_mHealth_ 2.55 [SD 1.24]; Mean_general_ 1.66 [SD 89]), *t*_310_=31.21, *P*<.001. Family and friends are the most trusted and preferred source of information. The two groups differ significantly regarding the use of the Internet: χ^2^_1_=22.4, *P*<.001; app store: χ^2^_1_=5.3, *P*<.05; magazines: χ^2^_1_=25.3, *P*<.001; and television: χ^2^_1_=9.8, *P*<.05 as sources of information. Users of health apps significantly more often reported the mentioned sources as relevant for them.

For the sources of information, associations with demographic characteristics were also calculated to identify influencing factors (see Table 7). Family and friends as source of information was associated with young age and high technical readiness and high computer literacy (AOR=0.96 [95% CI 0.92-0.99] for age, AOR=1.43 [95% CI 0.99-2.04] for technical readiness, and AOR=1.1 [95% CI 1.04-1.17] for computer literacy).

**Table 4:** Mentioned reasons decreasing subjective acceptance of health apps (multiple answers allowed).

Reasons		User group		Significance
		Health app users	General app users	
Number of reasons mentioned, mean (SD)		1.23 (0.98)	1.45 (0.95)	*t*_310_=25.37, *P*<.001
**Mentioned reasons, n (%)**				
	Lack of trust	62 (65.3)	157 (72.7)	χ^2^_2_=1.7, *P*=.19
	Data privacy concerns	27 (28.4)	63 (29.2)	χ^2^_2_=0.0, *P*=.89
	Fear of misdiagnosis	20 (21.1)	33 (15.3)	χ^2^_2_=1.5, *P*=.21
	Poor usability	6 (6.3)	33 (15.3)	χ^2^_2_=4.8, *P*=.02
	Lack of self-confidence	1 (1.0)	15 (6.9)	χ^2^_2_=4.6, *P*=.03
	Lack of interest^a^	1 (1.0)	12 (5.6)	χ^2^_2_=3.7, *P*=.05
	Pressure to perform^a^	0 (0)	2 (0.9)	χ^2^_2_=3.7, *P*=.05
	Technical reasons^a^	0 (0)	2 (0.9)	χ^2^_2_=3.7, *P*=.05

^a^Answers to open-ended answer option; coded for analysis.

**Table 5:** Association between demographic characteristics and selected reasons for a lack of acceptance of health apps is shown.

Demographic characteristics	Reason									
	Lack of trust (n=184), AOR^a^ (95% CI)	*P* value	Data privacy concerns (n=81), AOR (95% CI)	*P* value	Fear of misdiagnosis (n=45), AOR (95% CI)	*P* value	Poor usability (n=36), AOR (95% CI)	*P* value	Lack of self- confidence (n=19), AOR (95% CI)	*P* value
Age	0.97 (0.93-1.02)	.21	.98 (0.93-1.03)	.41	0.92 (0.85-0.99)	.03	1.08 (1.01-1.16)	.03	0.98 (0.89-1.08)	.69
Education	—	.70	—	.83	—	.37	—	.38	—	.77
Multimorbidity	1.19 (0.98-1.45)	.07	1.19 (0.94-1.5)	.14	1.42 (1.07-1.89)	.02	0.987 (0.7-1.4)	.94	1.32 (0.85-2.05)	.22
Health competence	0.98 (0.89-1.07)	.65	0.93 (0.83-1.04)	.20	0.93 (0.81-1.07)	.29	0.8 (0.69-0.92)	.01	0.66 (0.53-0.82)	>.001
Technical readiness	1.74 (1.18-2.58)	.01	1.66 (1.01-2.71)	.04	1.94 (1.01-3.73)	.04	0.41 (0.21-0.78)	.01	0.32 (0.13-0.77)	.01
Computer literacy	1.08 (1.02-1.14)	.01	1.11 (1.03-1.21)	.01	1.08 (0.98-1.19)	.13	1.09 (0.99-1.2)	.08	0.99 (0.88-1.13)	.91

^a^AOR: adjusted odds ratio.

**Table 6:** Source of information participants rely on regarding apps (multiple answers allowed).

Sources		User group		Significance
		Health app users	General app users	
Number of sources used, mean (SD)		2.55 (1.24)	1.66 (0.89)	*t*_310_=31.21, *P*<.001
**Type of Sources, n (%)**				
	Family and friends	78 (82.1)	162 (75.0)	χ^2^_1_=1.8, *P*=.16
	Internet	52 (54.7)	58 (26.6)	χ^2^_1_=22.4, *P*<.001
	App store	47 (49.5)	77 (35.6)	χ^2^_1_=5.2, *P*<.05
	Magazines	40 (42.1)	34 (15.7)	χ^2^_1_=25.2, *P*<.001
	Television	15 (15.8)	11 (5.1)	χ^2^_1_=9.8, *P*<.05
	Experts	10 (10.5)	16 (7.4)	χ^2^_1_=0.8, *P*=.36

**Table 7:** Association between demographic characteristics and sources of information about apps.

Demographic characteristics	Source of information											
	Family and friends (n=203), AOR^a^ (95% CI)	*P* value	Internet (n=96), AOR (95% CI)	*P* value	App store, AOR (95% CI)	*P* value	Magazines, AOR (95% CI)	*P* value	Television, AOR (95% CI)	*P* value	Experts, AOR (95% CI)	*P* value
Age	0.96 (0.92-0.99)	.04	0.99 (0.94-1.05)	.87	0.94 (0.89-0.99)	.04	0.97 (0.91-1.04)	.37	0.88 (0.78-0.99)	.04	0.96 (0.87-1.05)	.33
Education	—	.16	—	.38	—	.08	—	.92	—	.94	—	.99
Multimorbidity	0.9 (0.74-1.08)	.25	1.2 (0.96-1.51)	.11	0.90 (0.72-1.13)	.4	0.87 (0.66-1.15)	.33	1.24 (0.8-1.93)	.33	1.13 (0.8-1.62)	.47
Health competence	0.94 (0.86-1.02)	.13	0.93 (0.84-1.02)	.13	0.93 (0.84-1.02)	.12	0.97 (0.86-1.08)	.56	1.01 (0.83-1.23)	.89	1.08 (0.91-1.28)	.36
Technical readiness	1.43 (0.99-2.04)	.05	3.36 (2.1-5.4)	>.001	3.11 (1.95-4.97)	>.001	3.24 (1.84-5.69)	>.001	3.22 (1.26-8.19)	.01	1.95 (0.93-4.09)	.08
Computer literacy	1.1 (1.04-1.17)	.03	1.11 (1.03-1.21)	.01	1.13 (1.05-1.22)	.001	1.13 (1.03-1.25)	.01	1.09 (0.93-1.29)	.26	1.04 (0.91-1.17)	.58

^a^AOR: adjusted odds ratio.

## Discussion

### Principal Findings

The potential of mobile phone apps to increase quality in health care and thus the QoL of patients is currently the topic of much discussion [3,11]. Initial studies have investigated the effects of smartphone apps on diabetes and chronic disease management, as well as fall risk [9-12]. The focus of this study lies on the investigation of the prevalence of health apps among older adults in Germany.

This explorative study presents the results of a postal survey. The response rate was about 11.52% (576/5000). The sample (N=576) was divided into three groups (users of health apps (n=95), users of general apps (n=216), and nonusers of apps (n=265). Most participants (54.0%, 311/576) use on average up to ten general apps and on a daily basis. Moreover, 16.5% (95/576) of the whole sample already use at least one health app. Participants most frequently reported having “1-5 health apps” installed on their smartphone and which they use on a weekly basis. Health apps were therefore found to be less frequently used than general apps (daily basis). Asked about the type of health app used, participants mentioned exercise apps most frequently (29%, 28/95), followed by rating apps (26%, 25/95), or apps for first aid response (13%, 12/95). Age and high technical readiness were positively associated with the use of health apps. The average number of chronic diseases and health competence as measured by an adapted version of the EU HLS-47 did not differ significantly between the three groups. Participants suffered on average from at least one chronic disease, whereby the most frequently reported diseases for all three groups were hypertension and back pain. Factors such as gender or education showed no significant differences between the three groups and thus, no influence on the use of health apps. Results about barriers of health app use showed that a lack of trust, data privacy concerns, and fear of misdiagnosis are the main ones for health as well as general app users. Differences between the two groups were revealed for poor usability (χ^2^_1_=4.8, *P*=.02) and a lack of self-confidence (χ^2^_1_=4.7, *P*=.03). These barriers were more frequently mentioned by general app users. These barriers are therefore possible reasons why general app users do not engage with health apps [5]. Finally participants were asked about their preferred source of information about apps. Users of health apps reported a significantly higher number of sources than users who only use general apps (Mean_mHealth_ 2.55 [SD 1.24] and Mean_general_ 1.66 [SD 0.89]). The most frequent source participants rely on is family and friends. Significant differences were revealed for sources such as Internet, app store, magazines, and television, which were more frequently mentioned by health app users than general app users. Experts were the least frequently mentioned source of information. Therefore, targeting older adults’ family and friends as motivators for health app use seems to be a promising approach.

Our study adds to existing research by reporting associated factors of health app use, frequency of use, as well as source of information regarding apps for older adults in Germany. In contrast to findings in previous national surveys in the United States and Hong Kong, older German adults are likely to use health apps if they are at the lower end of the age range and have a high level of technical readiness [13,14]. About 16.5% of questioned older adults already used at least one health app, which is comparable with the national survey results for populations in the United States and Hong Kong [13,14]. These results can be considered as reliable, as the reported response rate of our survey (11.52%, 576/5000) is consistent with methodical investigations of response rates among older adults [18]. Regarding health app use, our findings extend current research results because besides the simple possession of health apps, we also investigated their use in terms of number of installed apps and frequency of use associated with type of app used among older adults in Germany.

Shen et al reported for the Hong Kong territory an association between history of chronic disease, as well as education and health app use [13]. In our study, neither association was confirmed. This might be related to the already high level of education within our sample. Comparing our participants’ demographics with demographics of the general population in Germany reveals a higher level of education and higher representation of male individuals in our sample than would be expected for the population of adults older than 60 years (high level of education: 38.5% sample vs 16.5% general population; male gender: 51% sample vs 43% general population) [1,24]. Further efforts are needed to verify our results in a sample with individuals with lower levels of education, as they might have poor health status and therefore, would profit most from such apps [6,13]. A way to reach these groups are family and friends, as these are the most frequently used source of information about apps and therefore, would promote this topic best.

Krebs and Duncan reported that the group of exercise-related health apps is the most frequently used one among adults in the United States [15]. Our study showed consistent results as exercise-related health apps were also mentioned most frequently in our study. Further studies need to identify whether exercise-related apps are the initial point of contact with health-related apps or not. This would allow developers of health apps to get more insights on how to design initial contact and what prerequisites they could expect if developing health apps for older adults.

Identified barriers in our study were, in order of frequency, a lack of trust, data privacy concerns, and fear of misdiagnosis. This is a contrast to findings of Krebs and Duncan, where lack of interest, high cost, and lack of trust in apps collecting their data were mentioned most [15]. The costs of health apps were not a reason for older German adults to avoid the use of health apps, maybe because of the fact that most health care-related costs are paid by the health insurance companies, and this would also be expected for future health apps.

### Limitations

This study has several limitations relating to its methodological design as well as the reported results. The postal study was not representative because of its self-selection. Although the selection of the original 5000 potential participants was adequate in terms of representative population characteristics, a bias is still possible as these decided by self-selection to take part in this study. A bias in recruitment will lead to differences for the groups in the use of health apps as well as in age and education. Due to the postal recruitment method, no inferences can be made about the frequency of use and sociodemographic distribution of the general user group of health apps, especially as this postal survey was conducted with older adults in Germany. Finally, it must be noted that we are unable to answer how strongly participants are engaged with their health apps in terms of time of use and adherence [25]. Although we know how frequently they consult their health apps, we did not investigate which specific tasks they use them for.

### Conclusions

In an exploratory approach, we investigated the prevalence of health apps among older adults in Germany. A prevalence of health apps was identified, with exercise-related health apps being the most frequently used ones. We were able to determine barriers to and incentives for health app use and compared these with recent results from the United States and Hong Kong.

## Appendix

Multimedia Appendix 1 Introduction text of survey in German and English.

## Abbreviations

AOR: adjusted odds ratio
EU HLS-47: European Health Literacy Survey-47
QoL: quality of life
