# Supplementary material for: Prevalence of Health App Use Among Older Adults in Germany: National Survey
Source: JMIR Mhealth Uhealth. 2018 Jan 23;6(1):e26. doi: 10.2196/mhealth.8619 (PMC5801520; doi:10.2196/mhealth.8619)
Supplement: Multimedia Appendix 1 [file mhealth_v6i1e26_app1.pdf]

Introduction text of survey (German)

Forschen Sie mit uns für eine bessere Zukunft in der Gesundheitsversorgung

Sehr geehrte Frau/sehr geehrter Herr X,

mit dem beiliegenden Fragebogen möchten wir Sie um Ihre Mithilfe in unserem gemeinsam mit dem Bundesministerium für Bildung und Forschung durchgeführten Forschungsprojekt bitten. Im Rahmen unseres Projekts untersuchen wir, wie digitale Produkte von Ihnen genutzt werden und in welchem Zusammenhang Sie deren Benutzung befürworten.

Wir würden uns freuen, wenn Sie an unserer Befragung teilnehmen und damit uns und das Ministerium dabei unterstützen, die Zukunft der künftig zunehmend digitalen Gesundheitsversorgung in Deutschland sinnvoll zu gestalten.

Um diese Umfrage mit so wenig Aufwand wie möglich für Sie zu verbinden, finden Sie beiliegend einen Rückumschlag für den Fragebogen. Damit können Sie diesen bequem über jeden Briefkasten der Deutschen Post kostenlos an uns zurückschicken.

Ihre Antworten werden im Sinne guter wissenschaftlicher Praxis von uns vollkommen anonym behandelt und werden nicht an Firmen oder Dritte weitergegeben.

Wichtig bei der Beantwortung der Fragen ist, dass es keine richtige oder falsche Antwort gibt. Antworten Sie entsprechend Ihrer Ideen und Überzeugung. Für Rückfragen oder weitere Informationen zu diesem Forschungsprojekt stehen wir Ihnen gerne telefonisch oder per E-Mail zur Verfügung.

Wir bedanken uns für Ihre Teilnahme an unserem Forschungsprojekt und Ihre Unterstützung zur Gestaltung der Zukunft des deutschen Gesundheitssystems und verbleiben mit freundlichen Grüßen,

Forschungsgruppe Tech4Age

Introduction text of survey (English translation)

Research with us for a better future in health care

Dear Mrs. X / Mr. X,

With the enclosed questionnaire, we would like to ask you for your assistance in our research project conducted together with the Federal Ministry of Education and Research of Germany. Within the framework of our project, we are investigating how digital products are used by you and in what context you advocate their use.

We would be very pleased if you would participate in our survey and thus help us and the Ministry to make the future of the increasingly digital health care in Germany meaningful.

In order to keep things simple for you, you will find enclosed a return envelope for the questionnaire. This allows you to send it back to us free of charge.

Your answers will be treated completely anonymously by us in the sense of good scientific practice and will not be passed on to companies or third parties.

There is no right or wrong answer. Respond to your ideas and beliefs. Please do not hesitate to contact us by phone or e-mail for assistance or further information on this research project.

We would like to thank you for your participation in our research project and your support for shaping the future of the German health-care system and remain with kind regards,

Research Group Tech4Age
